# The effect of the first office blood pressure reading on hypertension-related clinical decisions

**DOI:** 10.5830/CVJA-2012-052

**Published:** 2012-09

**Authors:** Idris Oladipo, Adedokun Ayoade

**Affiliations:** Department of Family Medicine, Lagos State University Teaching Hospital (LASUTH), Ikeja, Lagos, Nigeria; Department of Family Medicine, Lagos State University Teaching Hospital (LASUTH), Ikeja, Lagos, Nigeria

**Keywords:** first BP reading, hypertension, clinical decisions

## Abstract

**Abstract:**

The effect of the first office blood pressure reading (FBPR) on hypertension-related decisions was evaluated using blood pressure (BP) readings taken with the BpTRU BPM-100 device. BP readings were grouped into three pairs: (1) single readings (first and second readings), (2) computed average of three readings (one including and one excluding the first reading), and (3) computed average of five readings (one including and one excluding the first reading). Categorisation of BP readings under JNC-7 classes and distribution into < 140/90 and ≥ 140/90 mmHg groups were selected as parameters guiding hypertension-related decisions. Readings including FBPR had strong positive correlations to those excluding FBPR (Pearson’s correlation coefficient ranged from 0.86–1.00). Also, FBPR-included and FBPR-excluded readings did not differ statistically in JNC-7 categorisation or distribution into < 140/90 or ≥ 140/90 mmHg groups. Our findings suggest that exclusion of FBPR may have no significant impact on hypertension-related clinical decisions.

## Abstract

Issues regarding the validity and reliability of office blood pressure (OBP) readings have challenged the prime role hitherto played by OBP measurements in the management of hypertension. The white-coat effect, masked hypertension and various observer biases are the chief factors compromising the usefulness of OBP readings.^1^ The result has been a shift to the use of ‘out of office measurements’ such as home blood pressure measurements (HBPM) and ambulatory blood pressure measurements (ABPM) as more reliable assessors of blood pressure (BP).^2^ HBPM has also been shown to be a better predictor of cardiovascular risk than OBP.^3^

The wide applicability of ABPM is greatly limited by the high cost of this technology. At present, it is not feasible to have all patients conduct HBPM prior to hospital visits, especially among resource-poor populations. HBPM is challenging for the visually impaired and the elderly with psychomotor impairments. The diversity in the design of devices used in HBPM and variability in their algorithms and outputs continue to give cause for concern. There will be a continued need for clinicians to conduct OBP measurements. Therefore efforts geared towards the improvement of the validity and reliability of OBP measurements would be invaluable.

For clinical decisions, most guidelines recommend the use of average BP values derived from multiple readings. This is to achieve a closer approximation of BP readings to the patient’s true BP by compensating for the intrinsic physiological variability of BP with each heart beat (the beat-to-beat variation of BP).^4^,^5^ However, the constraints of time and limited availability of trained personnel have sustained the practice of taking a single measurement in the waiting room or the doctor’s office. This is particularly common in busy clinics serving resource-deficient settings.

Discarding the first blood pressure reading (FBPR) and using the average of the next two or more readings has also been advanced as a strategy to improve the accuracy of BP readings. One important reason cited for the exclusion of FBPR is the theoretical potential of this strategy to compensate for the ‘office pressor effect’ – a phenomenon characterised by the recording of a high first BP reading that is followed by lower BP readings.^6^ While the beat-to-beat variability of blood pressure and the white-coat phenomenon justify the need for multiple readings and use of mean BP values, the additional benefit of discarding the first reading has not been proven.

Blood pressure-related clinical decisions are based on a synthesis of several clinical parameters. One such is the categorisation of the patient’s BP reading on a reference classification system. The BP classification published in the 7th report of the Joint National Committee on the prevention, detection, evaluation and treatment of hypertension (JNC-7 classification) is the most recent and most widely used.^7^ The localisation of the blood pressure reading relative to a threshold value (e.g. < 140/90 mmHg for control in patients with uncomplicated hypertension or ≥ 140/90 mmHg for diagnosis of hypertension in the office setting) is another important parameter that influences the clinician’s decisions.

Despite being advocated as a beneficial clinical practice, the value of discarding the first blood pressure reading (FBPR) is yet to be determined by research. In this study, we aimed to explore the impact of FBPR on hypertension-related clinical decisions in a general out-patient setting. Our objectives were to evaluate the impact of FBPR on (1) the distribution of participants’ BP readings using the JNC-7 classification model and a customised modification, (2) consideration of a diagnosis of hypertension among the previously undiagnosed sub-population of the study sample, (3) clinical assessment of BP control among the previously diagnosed and treated hypertensive sub-group of the participants.

## Methods

This descriptive, cross-sectional study was conducted among a selected sample of 186 consenting adults (aged 18 years and over) attending the general out-patients’ clinic of the Department of Family Medicine, Lagos State University Hospital (LASUTH), Lagos, Nigeria. The general out-patients’ clinic is the first contact clinic for all patients presenting at the hospital with non-urgent conditions. The study was approved by the Institution’s research ethics committee (LHREC/08/054).

Participants were consecutively recruited in the waiting hall of the clinic, where patients are seated and take their turn to see the nurse for evaluation of vital signs. The study was introduced to each patient by the clinic nurse, who offered each adult the option of having automated blood pressure measurement with the use of a device that could obtain six consecutive readings in a designated office. Associated time required and attendant discomfort from repeated cuff inflation and deflation were explained to prospective participants. Signed informed consent was obtained from all individuals who agreed to participate.

Inclusion criteria were: willingness to voluntarily participate and the absence of any acutely distressful condition such as fever, breathlessness and pain. Patients excluded were those with irregular pulse rhythm or a mid-upper arm circumference greater than 42 cm (oscillometric devices are unreliable in persons with arrhythmias or mid-upper arm circumference > 42 cm), and all patients who had smoked cigarettes or taken coffee on the day of examination.^4^

Blood pressure measurements were conducted in an office devoid of noise or vibrations, offering optimum comfort to the participants. Patients were instructed by a trained research assistant to relax and avoid arm movement during the measurements. The research assistant witnessed and documented the reading obtained from the first measurement, and then left the room. Participants were trained and instructed to remove the cuff and press a door bell, calling for the return of the research assistant after the completion of the six measurements. All measurements were taken with an automated oscillometric blood pressure machine, BpTRU BPM-100 (VSM Medical Technologies, Canada) with strict adherence to the American Heart Association recommendations for clinical BP measurement.^4^

The BpTRU machine uses an oscillometric algorithm for the determination of systolic and diastolic BP. It is designed to take six BP readings with a programmable rest interval between each measurement (resting time between measurements can be set at one, two or three minutes). The interval between measurements has been shown to have no effect on the readings obtained by this device.^8^ For this study, the resting time was set at one minute to keep the time committed to the study by each volunteer to the lowest possible, in order to minimise the interference of the study with their primary aim of clinic attendance.

The BpTRU device automatically discards the first reading and computes the average systolic and diastolic BP from the average of the last five readings. All six readings (including the first reading) as well as the computed average are digitally displayed. The device has been validated with the British Hypertension Society (BHS) protocol and passed with an A/A grade.^9^

All the BP readings displayed by the machine were accurately documented. The average readings computed by the device were also recorded. Participants’ biodata, history of hypertension/use of antihypertensive medications were also noted. Data collection was concluded on the 186th participant (after six months) due to resource (time, space and personnel) constraints. At this point, it was judged that the sample would suffice for the purpose of the study because adequate representation of hypertensive and non-hypertensive participants had been attained to ensure adequate statistical power for sub-population analysis. The final study sample was separated into two sub-populations, namely, a hypertensive sub-population (individuals with a previous diagnosis of hypertension) and a mixed sub-population comprising normotensive and yet undiagnosed possibly hypertensive individuals.

Three BP measurement models, namely single, triple (average of three consecutive readings) and quintuple (average of five consecutive readings) BP readings were created from the database. We evaluated the effect of inclusion or exclusion of FBPR on: (1) distribution of participants’ BP readings in a JNC-7 classification model for the mixed sub-population and a modified JNC-7 classification model (in which the optimal BP and pre-hypertensive domains were merged and relabeled stage 0) for the hypertensive sub-population, (2) consideration of a diagnosis of hypertension among the mixed sub-population of the study sample, and (3) clinical assessment of BP control among the hypertensive sub-population of the participants. The correlations between the readings that included FBPR and those excluding FBPR were also evaluated.

Lastly, differences between the compared readings in each measurement model were evaluated to determine and compare the proportion of differences among the hypertensive and mixed sub-populations respectively, those = 0 mmHg and those ≥ 5 mmHg. (Differences ≥ 5 mmHg are considered clinically significantly different in the British Hypertension Society’s protocol for validating BP devices).^10^

Variables used in data analysis included:

• first BP reading (SYS-1 and DIA-1)
• second BP reading (SYS-2 and DIA-2)
• average of three readings, including FBPR: SYS_1-3_ and DIA_1-3_
• average of three readings, including FBPR: SYS_2-4_ and DIA_2-4_
• average of five readings, including FBPR: SYS_1-5_ and DIA_1-5_
• average of five readings, including FBPR: SYS_2-6_ and DIA_2-6_.

Data redesignation became imperative because of the need for joint consideration of systolic and diastolic values for JNC classification and relativity to the threshold of 140/90 mmHg. SYS_1-3_/DIA_1-3_, SYS_2-4_/DIA_2-4_, SYS_1-5_/DIA_1-5_ and SYS_2-6_/ DIA_2-6_ were designated as AVE_1-3_, AVE_2-4_, AVE_1-5_ and AVE_2-6_, respectively. Likewise, SYS-1/DIA-1 and SYS-2/DIA-2 were designated as FBPR and SBPR, respectively. Cases in which systolic and diastolic readings fell under different JNC-7 stages were treated by classifying the BP reading under the higher category, as recommended in the JNC-7 report. This principle was extended to the classification of the BP readings into < 140/90 or ≥ 140/90 mmHg.

## Statistical analysis

Data analysis was performed using GraphPad Prisms version 5 for Windows. All study data were first evaluated with descriptive statistics. In addition to the final study sample, sub-groups that included the hypertensive sub-population (those who had been diagnosed as having hypertension) and a mixed sub-population (comprising true normotensives and undiagnosed hypertensives) were identified, analysed and compared.

Correlation statistics and comparison of mean values were performed after evaluation of data for normality. Comparisons involved the pairs of: FBPR and SBPR, AVE_1-3_ and AVE_2-4_, and AVE_1-5_ and AVE_2-6_. Differences in the distribution of compared BP readings under the JNC-7 model for the mixed sub-population and a modified JNC-7 classification model for the hypertensive sub-population were respectively tested for significance using the Chi-square test.

Fisher’s exact test was used in evaluating the statistical significance of the differences in the distribution of compared readings under the < 140/90 mmHg and ≥ 140/90 mmHg groups. Correlation between the compared variables was evaluated using Pearson’s correlation. For all statistical tests, a *p*-value < 0.05 was considered statistically significant.

## Results

Blood pressure measurements were conducted on 186 consenting adults. Of these, 170 participants (91.4%) had complete sets of the six readings required for data analysis. This was taken as the final study population. This final study sample comprised BP readings from 87 males and 83 females (M:F = 1.05:1), with age range 18–86 years (46.7 ± 13.9).

Patients who had been previously diagnosed as having systemic hypertension comprised 35.9% (*n* = 61) of the final sample. All had received prescription(s) for antihypertensive medications during previous clinic visits. Females comprised 59% (*n* = 36) of the hypertensive sub-population and 43% (47 of 109) of the remaining mixed sub-population (*p* = 0.06, Fisher’s exact test). Patients comprising the hypertensive sub-population were older than those in the mixed sub-population [24–86 years (51.3 ± 12.2) vs 18–74 years (44.1 ± 14.2); *p* = 0.0009, independent *t*-test].

Table 1 shows the descriptive statistics for the BP variables that were compared in this study. These were presented as range of values (mean ± standard deviation). Within each of the groups in Table 1, the independent *t*-test found statistically non-significant differences (in respective systolic and diastolic component comparison) between the means of the first and second BP readings, as well as the means of the averages of three readings, and the averages of five readings (*p* > 0.05 for all).

**Table 1.** Blood Pressure Variables Involved In The Comparative Analyses

*Variables*	*Final study population n = 170 (mmHg)*	*Hypertensive sub-population n = 61 (mmHg)*	*Mixed sub-population n = 109 (mmHg)*
SYS-1	93–244 (146 ± 32.4)	112–244 (164.9 ± 32.2)	93–228 (135.5 ± 27.4)
DIA-1	44–143 (86.3 ± 17.3)	44–143 (95.4 ± 15.4)	55–136 (81.2 ± 16.2)
SYS-2	87–250 (141.8 ± 31.1)	105–250 (159.7 ± 31.8)	87–225 (131.8 ± 25.8)
DIA-2	51–143 (83.9 ± 16.4)	60–143 (92.6 ± 14.3)	51–129 (79.1 ± 15.5)
SYS_1-3_	90–248 (141.9 ± 31.1)	109–249 (160.3 ± 31.4)	90–223 (131.6 ± 25.9)
DIA_1-3_	52–143 (84.1 ± 16.6)	59–143 (93.1 ± 14.5)	52–130 (79.1 ± 15.7)
SYS_2-4_	88–250 (138.4 ± 30.4)	100–250 (156 ± 31.2)	88–220 (128.6 ± 25.2)
DIA_2-4_	49–143 (82.3 ± 16.3)	54–143 (90.6 ± 14.3)	49–128 (77.7±15.6)
SYS_1-5_	89–249 (139.1 ± 30.4)	103–249 (156.9 ± 30.9)	89–219 (129.2 ± 25.2)
DIA_1-5_	49–143 (82.5 ± 16.3)	55–143 (91.1 ± 14.3)	50–128 (77.7 ± 15.4)
SYS_2-6_	87–250 (136.5 ± 29.8)	103–250 (153.9 ± 30.6)	87–220 (126.8 ± 24.5)
DIA_2-6_	49–142 (81.5 ± 16.6)	53–142 (90.1 ± 14.7)	49–126 (76.6 ± 15.7)

For the single, triple and quintuple measurement models respectively, systolic/diastolic readings, which included the FBPR, were higher than those that excluded the first reading in 64.2%/58.7%; 78%/68.8%; 82.6%/78.9% of readings among the mixed population. Similarly, corresponding proportions among the hypertensive population were 67.2%/70.5%; 82%/78.7%; 82%/85.2% of the systolic/diastolic readings.

Differences between the sub-populations in the relative proportion of FBPR-included readings which were higher than FBPR-excluded readings were statistically insignificant for both systolic and diastolic comparisons (Chi-square test, *p* > 0.05 for all). In both sub-populations, it was observed than the tendency to have higher systolic/diastolic readings with the inclusion of FBPR was amplified in the triple and quintuple measurement models.

Conversely, in each of the three measurement models, readings excluding FBPR were greater than those including FBPR in 27.5%/35.8% (single), 9.2%/8.3% (triple), 4.6%/6.4% (quintuple) of the systolic/diastolic readings among the mixed population. Corresponding findings among the hypertensive sub-population were 26.2%/23%; 14.8%/9.8% and 13.1%/6.6%.

Similarly, differences between the sub-populations in the relative proportion of FBPR-excluded readings which were higher than FBPR-included readings were statistically insignificant for both systolic and diastolic comparisons (Chi-square test, *p* > 0.05 for all). It was also observed than the tendency to have higher systolic/diastolic readings with the exclusion of FBPR was reduced in the triple and quintuple models in both sub-populations.

Table 2 shows the differences (expressed as absolute values) between the compared readings. Readings including FBPR were found to be equal to those excluding FBPR in 8.3–12.8%/5.5–22.9% and 6.6–13.1%/6.6–11.5% of systolic/diastolic readings among the mixed and hypertensive sub-populations, respectively. Differences between the proportions of equal readings in the sub-populations were not statistically significant (*p* > 0.05 for all). Clinically significant differences (≥ 5 mmHg) were observed between compared systolic/diastolic readings in 18.3–56.9%/0.9–26.6% and 26.2–65.6%/1.6–47.5% of readings among the mixed and hypertensive sub-populations respectively.

**Table 2.** Differences Between Compared Readings

*Differences*	Range (mean ± SD*) (mmHg)	*0 mmHg n (%)*	*< 5 mmHg n (%)*	*≥ 5 mmHg n (%)*
Mixed sub-population				
SYS-1 – SYS-2	0–31 (6.9 ± 5.9)	9 (8.3)	47 (43.1)	62 (56.9)
SYS_1-3_ – SYS_2-4_	0–11 (3.4 ± 2.7)	14 (12.8)	77 (70.6)	32 (29.4)
SYS_1-5_ – SYS_2-6_	0-11 (2.5 ± 2.0)	14 (12.8)	89 (81.7)	20 (18.3)
DIA-1 – DIA-2	0–16 (3.8 ± 3.1)	6 (5.5)	80 (73.4)	29 (26.6)
DIA_1-3_ – DIA_2-4_	0–6 (1.6 ± 1.4)	25 (22.9)	105 (96.3)	4 (3.7)
DIA_1-5_ – DIA_2-6_	0–21 (1.6 ± 2.1)	15 (13.8)	108 (99.1)	1 (0.9)
Hypertensive sub-population				
SYS-1 – SYS-2	0–30 (9 ± 7.5)	4 (6.6)	21 (34.4)	40 (65.6)
SYS_1-3_ – SYS_2-4_	0–20 (5 ± 3.8)	2 (3.3)	31 (50.8)	30 (49.2)
SYS_1-5_ – SYS_2-6_	0–11 (3.3 ± 2.5)	8 (13.1)	45 (73.8)	16 (26.2)
DIA-1 – DIA-2	0–24 (5.1 ± 4.5)	4 (6.6)	32 (52.5)	29 (47.5)
DIA_1-3_ – DIA_2-4_	0–34 (3 ± 4.4)	7 (11.5)	54 (88.5)	7 (11.5)
DIA_1-5_ – DIA_2-6_	0–19 (1.9 ± 3.4)	5 (8.2)	60 (98.4)	1 (1.6)

The proportion of clinically significant differences between readings including FBPR and those excluding FBPR reduced greatly in the average measurement models. For each measurement model, the hypertensive sub-population had higher proportions of clinically significant differences (≥ 5 mmHg) between the compared readings than the mixed sub-population. However, the differences between the sub-populations in the proportion of differences (≥ 5 mmHg) were not statistically significant.

**Fig. 1.**
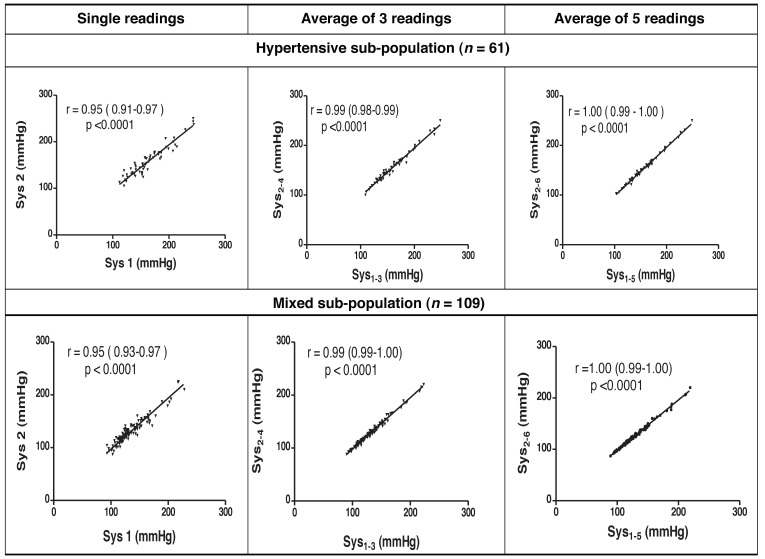
Correlation scatter plots and coefficients for compared systolic readings.

**Fig. 2.**
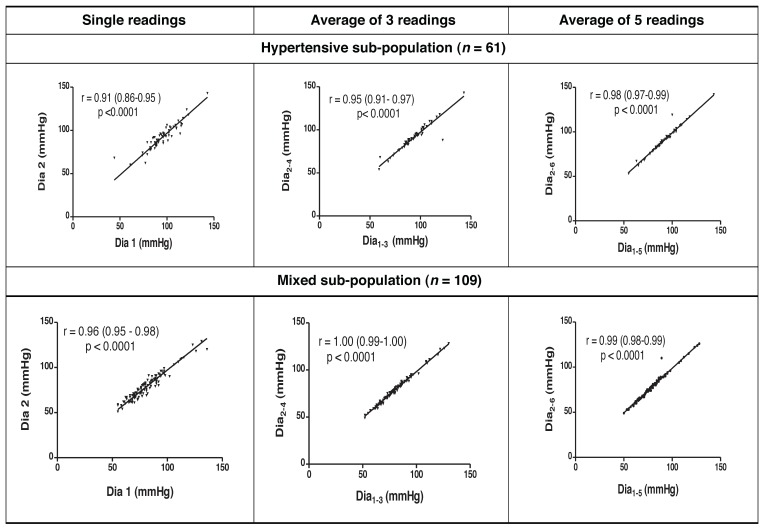
Correlation scatter plots and coefficients for compared diastolic readings.

Systolic and diastolic readings that included FBPR had strong statistically significant correlations to those excluding FBPR [Pearson’s correlation coefficient (*r*) 0.86–1.00, *p* < 0.0001 for all pairs of comparisons]. Scatter plots depicting these strong correlations as well as their coefficients (and 95% confidence intervals for the correlation coefficients) are shown in Figs 1 and 2 for systolic and diastolic readings, respectively.

The distributions of the subjects’ BP in the JNC-7 model (mixed sub-population) and a modified JNC-7 model (hypertensive sub-population) are shown in Table 3. No statistically significant difference was found in the pattern of distribution between the readings in all the comparisons within each sub-group.

**Table 3.** Distribution Of Blood Pressure Readings From Compared Variables Using Classical And Modified JNC-7 Models

*Variables*	*Optimal*	*Pre-HTN*	*Stage I*	*Stage II*	*ISH*	*χ^2^ (4 df)*	p*-value*
Mixed sub-population (*n* = 109)							
FBPR	37	30	14	14	14	1.37	0.84
SBPR	43	28	11	16	11		
AVE_1–3_	41	34	12	10	12	2.10	0.72
AVE_2–4_	45	37	6	10	11		
AVE_1–5_	43	37	9	9	11	0.45	0.93
AVE_2–6_	48	35	7	10	9		
*Variables*	*Stage 0*		*Stage I*	*Stage II*	*ISH*	*χ^2^ (3 df)*	p-*value*
Hypertensive sub-population (modified JNC-7 model) (*n* = 61)							
FBPR	11		9	30	11	0.37	0.95
SBPR	13		9	27	12		
AVE_1–3_	13		11	25	12	1.02	0.80
AVE_2–4_	16		8	23	14		
AVE_1–5_	17		11	21	12	0.46	0.93
AVE_2–6_	16		11	19	15		

Pre-HTN = pre-hypertension; ISH = isolated systolic hypertension, χ^2^ (4 df) = Chi-square test with 4 degrees of freedom, χ^2^ (3 df) = Chi-square test with 3 degrees of freedom.

Table 4 shows the changes in the distribution of subjects’ BP relative to a threshold value of 140/90 mmHg. It was observed that in the final study population, hypertensive sub-population and mixed sub-population, respectively, non-statistically significant differences were obtained in the distribution of BP values into < 140/90 mmHg and ≥ 140/90 mmHg groups by readings inclusive or exclusive of FBPR (*p* > 0.05 for all, Fisher’s exact tests).

**Table 4.** Comparison Of The Distribution Of Blood Pressure Readings Relative To A Threshold Value Of 140/90 mmHg

*Variables*	*<140/90 mmHg*	*≥ 140/90 mmHg*	p-value*
Final study population (*n* = 170)			
FBPR	79	91	
SBPR	86	84	0.52
AVE_1–3_	88	82	
AVE_2–4_	97	73	0.38
AVE_1–5_	96	74	
AVE_2–6_	100	70	0.74
Mixed sub-population (*n* = 109)			
FBPR	67	42	
SBPR	71	38	0.67
AVE_1–3_	75	34	
AVE_2–4_	82	27	0.37
AVE_1–5_	80	29	0.76
AVE_2–6_	83	26	
	*Controlled*		*Uncontrolled*
Hypertensive sub-population (*n* = 61)			
FBPR	11	50	0.82
SBPR	13	48	
AVE_1–3_	13	48	0.67
AVE_2–4_	16	45	
AVE_1–5_	17	44	1.00
AVE_2–6_	16	45	

*Fisher’s exact test.

## Discussion

In this study, comparative analysis was used to evaluate the effect of the first office BP reading on hypertension-related clinical decisions using single, triple and quintuple measurement models. Our results showed that mean readings that included or excluded FBPR (systolic and diastolic, respectively) within the final study population and the sub-populations did not differ in statistical significance.

The distribution of blood pressure readings for the hypertensive and mixed sub-populations in the classes defined by the JNC-7 model or its modification did not reveal any statistically significant difference relating to inclusion or exclusion of the FBPR for single, triple or quintuple measurements. Similarly, the distribution of the analysed blood pressure readings relative to a threshold of 140/90 mmHg did not differ significantly between readings that included or excluded FBPR.

A non-statistically significant difference was found between the hypertensive and mixed sub-populations in the comparison of the proportions of differences between readings that included and excluded the FBPR, which were: clinically significant (≥ 5 mmHg); and 0 mmHg. Lastly, we found that readings that included the FBPR were strongly correlated to those excluding the FBPR.

Overall, our findings suggest that: (1) for patient populations with known hypertensive status, the use of FBPR as a single reading or its inclusion in repeated readings for deriving average BP values may not have a significant effect on clinical decisions regarding BP control or staging of current hypertensive status for uncontrolled individuals; (2) for patient sub-populations with undetermined hypertensive status (commonly encountered in general outpatient clinics), the use of FBPR as a single reading or its inclusion in repeated readings for deriving average BP values may not have a significant effect on clinical decisions regarding BP classification or consideration for a diagnosis of hypertension; (3) in terms of clinically significant absolute differences (≥ 5 mmHg) between readings including and excluding FBPR, differences between a purely hypertensive population and a mixed population may not be of statistical significance. This suggests that a recommendation for the exclusion of FBPR for either of the population groups may not be clinically useful.

We observed that the issue of whether or not to discard the FBPR is an important one that has not received adequate research attention. Considering the fact that blood pressure measurement is associated with transient discomfort to the patient, subjecting patients to uncomfortable (albeit transient) cuff inflation and deflation to obtain a reading that will be discarded without a scientifically sound reason is unjustifiable. Discarding the FBPR is associated with the expending of patient and personnel time as well as energy. The consequence of this will be particularly relevant in resource-poor settings.

Graves and Grossardt had earlier found that discarding the first of three nurse-auscultatory or oscillometric blood pressure measurements did not improve the association of office blood pressure with ambulatory blood pressure readings.^11^ Despite conducting an extensive literature review, we were unable to identify any previous study that addressed this issue with sets of readings from the same BP monitor. However, it is noteworthy to state that Mengden *et al*. were the first to report that patients are also inclined to discard the first reading in home monitoring of blood pressure.^12^ It is not unlikely that this practice may have been acquired by the patients from physicians or nurses.

In our study, we focused on a single oscillometric device, the BpTRU, because it was designed for automated exclusion of the first BP reading. The value of this BpTRU design in improving the association between OBP and ABPM has been reported by Beckett and Godwin.^13^ Their findings suggested that by discarding the first reading, the improvement resulted from a reduction in the white-coat effect. Therefore, while this device may reduce the white-coat effect in comparative studies that involved other devices with different principles and algorithms, in our study, it has clearly been unable to justify the effect of discarding the first reading on important parameters that often guide hypertension-related clinical decisions.

We avoided the use of more than one device or additional auscultatory measurements because of confounding factors that could be introduced. Furthermore, it was considered that six BP readings at one sitting were taxing enough for our participants.

Important limitations of our study include the relatively small sample size. However, evaluation of our objectives in sub-populations showed that findings within the final study population were not likely due to chance. Also, these findings may not necessarily apply to automated oscillometric measurements in the home setting.

The strengths of this study lie in the wide range of blood pressure values that were involved and the use of a general out-patient population, which enabled us to perform sub-group analyses of hypertensive and undifferentiated sub-populations. There is a need for further studies with larger sample sizes to create more robust evidence on this important clinical subject. Also, investigation of the inclusion or exclusion of the first blood pressure reading in HBPM will illuminate its effect on the diagnosis and control of hypertension with the use of HBPM readings.

## Conclusion

Our findings suggest that exclusion of the first office BP reading is not likely to have a significant impact on hypertension-related clinical decisions.
